# Positive Feedback Organic‐on‐Silicon Upconversion Devices

**DOI:** 10.1002/advs.202511468

**Published:** 2025-11-09

**Authors:** Raju Lampande, Jon‐Paul DesOrmeaux, Adrian Pizano, Urcan Guler, John W. Hamer, Noel C. Giebink

**Affiliations:** ^1^ Department of Electrical Engineering and Computer Science University of Michigan Ann Arbor Michigan 48109 USA; ^2^ OLEDWorks LLC Rochester NY 14606 USA; ^3^ RTX Technology Research Center East Hartford Connecticut 06108 USA

**Keywords:** heterojunction photodiode, hybrid, OLED, positive feedback, upconverter

## Abstract

Positive feedback upconversion devices exhibit optoelectronic bistability and large photon‐to‐photon gain that can benefit applications ranging from night vision to neuromorphic image classification. Here, a hybrid device architecture is introduced that integrates a tandem organic light‐emitting diode (OLED) on top of an organic/Si heterojunction photodiode. These devices respond to near infrared light at wavelengths up λ ≈ 1.1 µm and exhibit a peak photon‐to‐photon upconversion gain of 9x when triggered with input light intensity <1 µW cm^−2^. It is found that minimizing the capacitance of the photodiode is important for maximizing the upconversion response speed of the device, and that electrical crosstalk in pixelated devices must be overcome to prevent cascading effects where one activated pixel triggers many others. These results mark an important step for optoelectronic upconverters that can be integrated with Si microelectronics, and they establish a path to extend the responsivity further into the near infrared by moving to other inorganic semiconductor platforms such as Ge or InGaAs.

## Graphical abstract

Positive optoelectronic feedback is achieved by depositing a tandem OLED on top of a heterojunction Si photodiode. The resulting hybrid device is bistable and upconverts near‐infrared light (λ = 1050 nm) to visible light (λ = 570 nm) with a photon‐to‐photon upconversion yield of up to 900%.

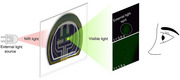

## 1 Introduction

Optoelectronic upconversion devices that amplify and convert near‐infrared (NIR) light directly into visible light are being explored for applications ranging from night vision^[^ ^1^ ^]^ to bioimaging.^[^ ^2^ ^]^ These devices typically consist of an organic light‐emitting diode (OLED) integrated back‐to‐back with an organic photodiode (OPD). Photocurrent generated by NIR light absorption in the reverse‐biased OPD flows through the forward‐biased OLED, leading to a proportional amount of visible light emission.^[^ ^3^, ^4^, ^5^ ^]^ Recently, we showed that using a multi‐stack tandem OLED in this device architecture can lead to large upconversion gain and bistability by establishing a positive feedback loop, where OLED light reabsorbed by the OPD results in photocurrent that leads to even more OLED emission in a self‐reinforcing cycle. Although all‐organic positive feedback upconversion devices are well‐suited for large area applications such as displays, a hybrid OLED‐on‐inorganic photodiode architecture could bring certain advantages, such as lower dark current (and thus higher sensitivity), extension of the spectral response further into the NIR, and monolithic integration with drive and control electronics.

Here, we demonstrate that positive feedback upconversion can be achieved in a hybrid device architecture where an organic‐Si p‐n heterojunction photodiode takes the place of the OPD. These devices respond to NIR light at wavelengths up λ ≈ 1.1 µm and exhibit a peak photon‐to‐photon upconversion efficiency η_pp_ >  9 when triggered with an input light intensity <1 µW cm^−2^. We find that minimizing the capacitance of the photodiode in the device is key to maximizing its response speed, and thus the frame rate that can be achieved for real‐time imaging in the positive feedback regime. Initial experiments on a pixelated array of 50x50 µm^2^ devices indicate that electrical crosstalk between neighboring pixels must be overcome to prevent cascading effects in which one activated pixel triggers many others. These results mark an important step for high gain upconversion devices that can be integrated with Si microelectronics, and they establish a clear path to extend the responsivity further into the NIR by moving to other inorganic semiconductor platforms such as Ge or InGaAs.^[^ ^6^, ^7^, ^8^, ^9^ ^]^

## 2 Results

### 2.1 Hybrid Upconverter Concept

**Figure 1:**
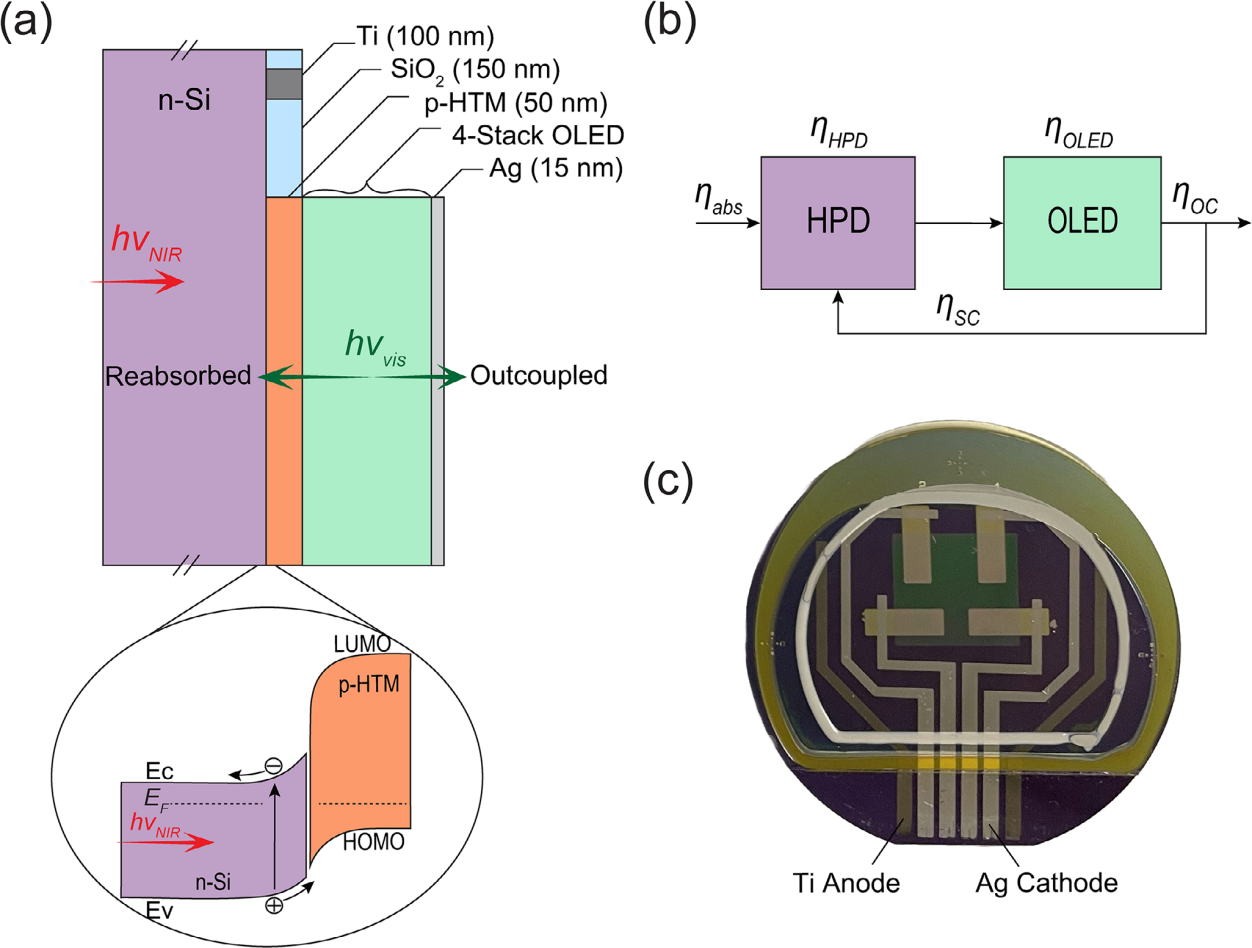
Device architecture. a) Schematic of the hybrid upconverter architecture. Ohmic Ti contacts are pre‐patterned on the n‐Si substrate via photolithography, and then the organic layer stack is deposited by vacuum thermal evaporation through an SiO_2_ window that defines the active device area. The Ag cathode contact is semitransparent to enable some of the OLED emission to be outcoupled in the forward direction. The inset highlights the p‐n heterojunction formed at the n‐Si/p‐HTM interface that enables photocurrent generation. b) Optoelectronic feedback loop that characterizes the operation of the device. A fraction (η_sc_) of the light emitted by the OLED is absorbed in the Si and generates photocurrent with internal quantum efficiency, η_HPD_. This photocurrent flows back through the OLED, which converts it to light with internal quantum efficiency η_OLED_. The fraction of OLED light outcoupled in the forward direction is η_oc_. c) Photograph of a typical sample with four active devices fabricated on a 2‐inch Si wafer. The gray and yellowish D‐shaped boundary regions are the liquid desiccant and encapsulation adhesive, respectively.

**Figure** **1**a illustrates the device architecture, where a 50 nm‐thick p‐doped organic hole transport material (HTM) is deposited on a 380 µm‐thick n‐type (As doped, ρ = 1 − 10 Ω‐cm) Si wafer followed by a green 4‐stack phosphorescent tandem OLED. The hybrid p‐n heterojunction formed between the n‐Si and p‐HTM (Figure 1a, inset) creates a depletion region near the Si surface, enabling photocurrent to be generated upon absorption of NIR light. As demonstrated in Ref. [10], when a photogenerated hole is injected into the tandem OLED, it produces up to four electroluminescent photons (one per OLED sub‐unit in the tandem stack), some of which can be reabsorbed by the hybrid photodiode (HPD, i.e., the photoactive Si/organic p‐n junction), initiating a positive feedback loop. Figure 1b illustrates this feedback schematically, where the loop gain, *f*, consists of the product of the HPD internal quantum efficiency (η_HPD_), the OLED internal quantum efficiency (η_OLED_), and the probability that green OLED‐emitted light is reabsorbed by the HPD (the self‐coupling efficiency, η_sc_). Positive feedback occurs when *f* = η_HPD_η_OLED_η_sc_ ≥ 1, which is possible because η_OLED_ exceeds unity. In the positive feedback regime, the device is bistable, with its low state set by the reverse saturation current of the HPD, and its high state limited by the resistance of the forward‐biased OLED. Compared to the OPD‐based positive feedback device from Ref. [10], the main advantages of moving to Si are extension of the spectral response further into the NIR (λ ∼ 1.1 µm) and the value of having a well‐developed platform for integration with the drive and control electronics needed for practical applications such as image intensification and optoelectronic neural network accelerators.^[^ ^11^ ^]^

### 2.2 Organic‐Si Heterojunction Photodiode

Since positive feedback operation hinges on the performance of the photodiode, it is important to characterize the operation of this sub‐component first. Silicon‐organic heterojunction diodes have been studied extensively in the past for photovoltaic applications,^[^ ^12^, ^13^ ^]^ in many cases using n‐Si and a degenerately‐doped p‐type organic semiconductor such as PEDOT:PSS.^[^ ^13^ ^]^ These studies indicate that the best HPD performance, characterized by low dark current and high photocurrent generation efficiency, is achieved by 1) minimizing the energetic offset between the Si valence band maximum and the organic semiconductor highest occupied molecular orbital (HOMO), and 2) ensuring that there is a large offset between the Si conduction band minimum and the organic lowest unoccupied molecular orbital (LUMO).^[^ ^12^ ^]^ A small valence band‐HOMO offset enables efficient collection of photogenerated holes, while a large conduction band‐LUMO offset provides a barrier to injection of majority electrons from the Si, thereby reducing the dark current.

**Figure 2:**
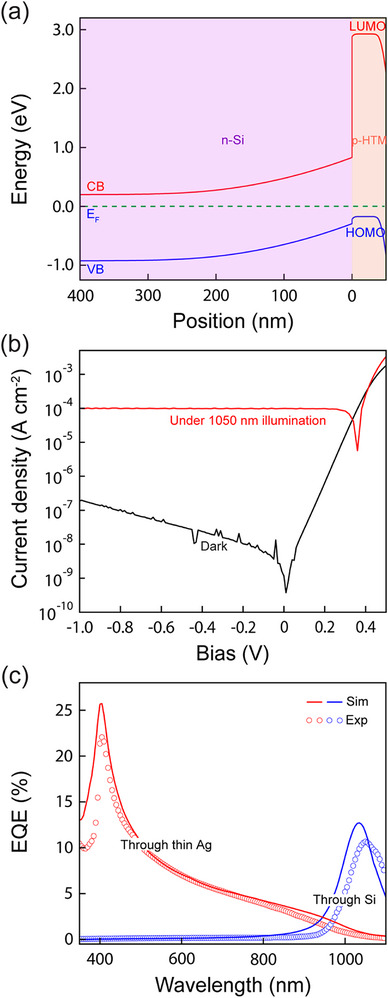
Hybrid photodiode performance. a) Simulated band diagram for the hybrid p‐n heterojunction at equilibrium, assuming vacuum level alignment with no interfacial dipoles. b) Current density‐voltage (J‐V) characteristics of the device measured in the dark and under illumination by a near‐infrared LED (λ = 1050 nm) with an intensity of 1.2 mW cm^−2^ incident through the Si substrate. Forward bias corresponds to positive voltage applied to the Ag contact in this measurement. c) Photocurrent external quantum efficiency (EQE) spectra collected in reverse bias for light incident through the Si substrate (blue), and through the semitransparent Ag top contact (red). The solid lines are the corresponding EQE curves predicted by a coupled optical and drift‐diffusion semiconductor device model using the commercial simulator SETFOS.

Based on these guidelines, we fabricate an HPD consisting of n‐Si/p‐doped HTM (50 nm, 6 vol% doping concentration)/MoO_3_ (5 nm)/Ag (15 nm). Although the HTM is a proprietary material, it is similar to 4,4′,4′'‐Tris[(3‐methylphenyl)phenylamino]triphenylamine (MTDATA), with an ionization potential of 5.1 eV and an electron affinity of ∼2.0 eV; the p‐dopant is similar to 2,3,5,6‐tetrafluoro‐7,7,8,8‐tetracyanoquinodimethane (F4‐TCNQ). The interfacial layer of MoO_3_ is included to help achieve an Ohmic contact for hole injection/extraction from the Ag. **Figure** **2**a shows the equilibrium band diagram simulated for this device based on estimated doping concentrations of *N*_d_ = 10^16^ cm^−3^ and *N*_a_ = 10^18^ cm^−3^ for the n‐Si and p‐HTM layers, respectively. This simulation highlights the large electron injection barrier formed by the p‐HTM layer and indicates a depletion width of roughly 0.2 µm in the near‐surface region of the Si.

Figure 2b shows the dark current‐voltage characteristic of the HPD, which exhibits an ideality factor of *n* ≈ 1.2 and stable reverse bias operation with a dark current density <200 nA cm^−2^ at *V*_a_ = −1 V. Illuminating the device with a near‐infrared LED (λ = 1050 nm) through the Si substrate yields a clear photovoltaic response with an open‐circuit voltage of ≈0.35 V. Figure 2c shows the external quantum efficiency (EQE) of the device illuminated through both the backside of the Si substrate and through the top semitransparent Ag contact. In the back side illumination case, the EQE peaks just above the Si band edge at λ = 1050 nm because absorption in the thick (i.e., most of the wafer thickness) quasi‐neutral region filters out all of the shorter wavelengths. In contrast, the front side‐illuminated EQE (through the Ag) peaks for short wavelength light since it is strongly absorbed in the depleted near‐surface region of the Si per Figure 2a. The sharp EQE decrease that occurs below λ ≈ 400 nm in this case is due to the absorption edge of the p‐HTM layer. Simulating the EQE from each side using a combined optical transfer matrix and drift‐diffusion electrical model qualitatively reproduces each data set, supporting this interpretation.

### 2.3 Positive Feedback Upconversion

**Figure 3:**
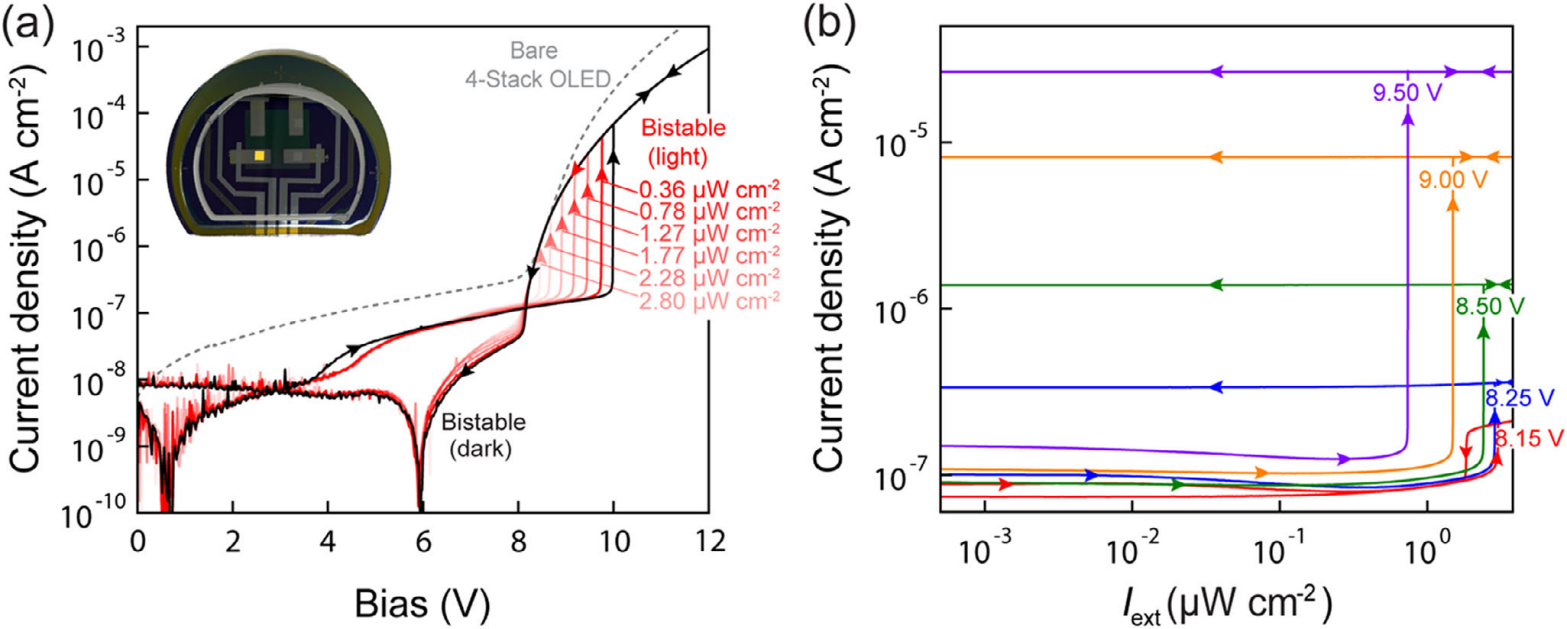
Positive feedback upconverter operation. a) Current density‐voltage characteristics of the upconversion device measured in the dark and under various levels of λ = 1050 nm external illumination incident through the Si substrate as shown in Supplementary Figure S1 (Supporting Information). The arrows indicate the voltage sweep direction with positive voltage applied to the Ti contact. The hysteresis loop in the 8 to 10 V range marks positive feedback operation; the low current hysteresis in the zero crossing is due to chemical capacitance that originates from charge trapping between the two back‐to‐back diodes that make up the device. The inset shows a photograph of a typical sample with one of the devices (the yellow square) activated in its high state at 9.7 V. b) Current density as a function of incident light intensity at several different fixed bias values, highlighting the <1 µW cm^−2^ threshold that triggers a jump from the lower to upper state of the device when *V*_a_ = 9.5 V. At this bias, the photon‐to‐photon upconversion gain for input light at the threshold intensity (≈0.86 µW cm^−2^) is η_pp_ = 2.1; it reaches a maximum of η_pp_ = 9 at higher bias as detailed in Supplementary Figure S2 (Supporting Information).

The full upconversion device is fabricated by depositing the 4‐stack green phosphorescent tandem OLED from Ref. [10] onto the HPD according to the structure in Figure 1a. **Figure** **3**a shows the J‐V characteristics of this device in the dark and under low levels of λ = 1050 nm LED illumination incident through the Si substrate. As with the original all‐organic upconverter in Ref. [10], we observe strong hysteresis and bistable behavior in the dark J‐V curve, confirming that the combination of self‐coupling, OLED, and HPD internal quantum efficiencies (η_sc_η_OLED_η_HPD_) in this device is sufficient to push the loop gain above unity and achieve positive feedback operation.

Hysteresis is similarly evident in the light input‐output characteristic (Figure 3b), where the device is operated at constant bias and the input illumination intensity is swept up and back down. When the input intensity reaches the ∼1 µW cm^−2^ threshold needed to achieve positive feedback on the up‐sweep (i.e., the increase in photocurrent flowing through the device increases η_OLED_ to the point at which *f* = 1), it triggers a jump from the lower to upper steady state, resulting in a sudden, hundred‐fold change in the output luminance of the device. The device then stays latched in its high state on the down‐sweep since the large amount of light emitted and reabsorbed internally ensures that there is enough photocurrent to maintain η_OLED_ above the threshold needed for *f* = 1.

One important difference between the all‐organic upconverter in Ref. [10] and the hybrid implementation here is that the J‐V curve in the former case is shifted to a higher voltage than the bare tandem OLED J‐V curve, whereas the two curves coincide in Figure 3a, at least at low current before the voltage drop from the large series resistance of the bulk Si wafer becomes significant. The absence of the voltage shift for the hybrid device reflects the fact that photogenerated charge in the Si depletion region is collected efficiently regardless of bias across the HPD (Figure 2b) whereas the organic PD in Ref. [10] required a substantial reverse bias to extract charge efficiently enough for the loop gain to reach unity. Because the hybrid upconverter requires less voltage to achieve the same high state current density, its luminous power efficiency is higher than the all‐organic device so long as their outcoupling efficiencies (η_oc_) remain similar. A table comparing the performance of the hybrid and all‐organic upconverter devices is provided in Supplementary Note S1 (Supporting Information).

**Figure 4:**
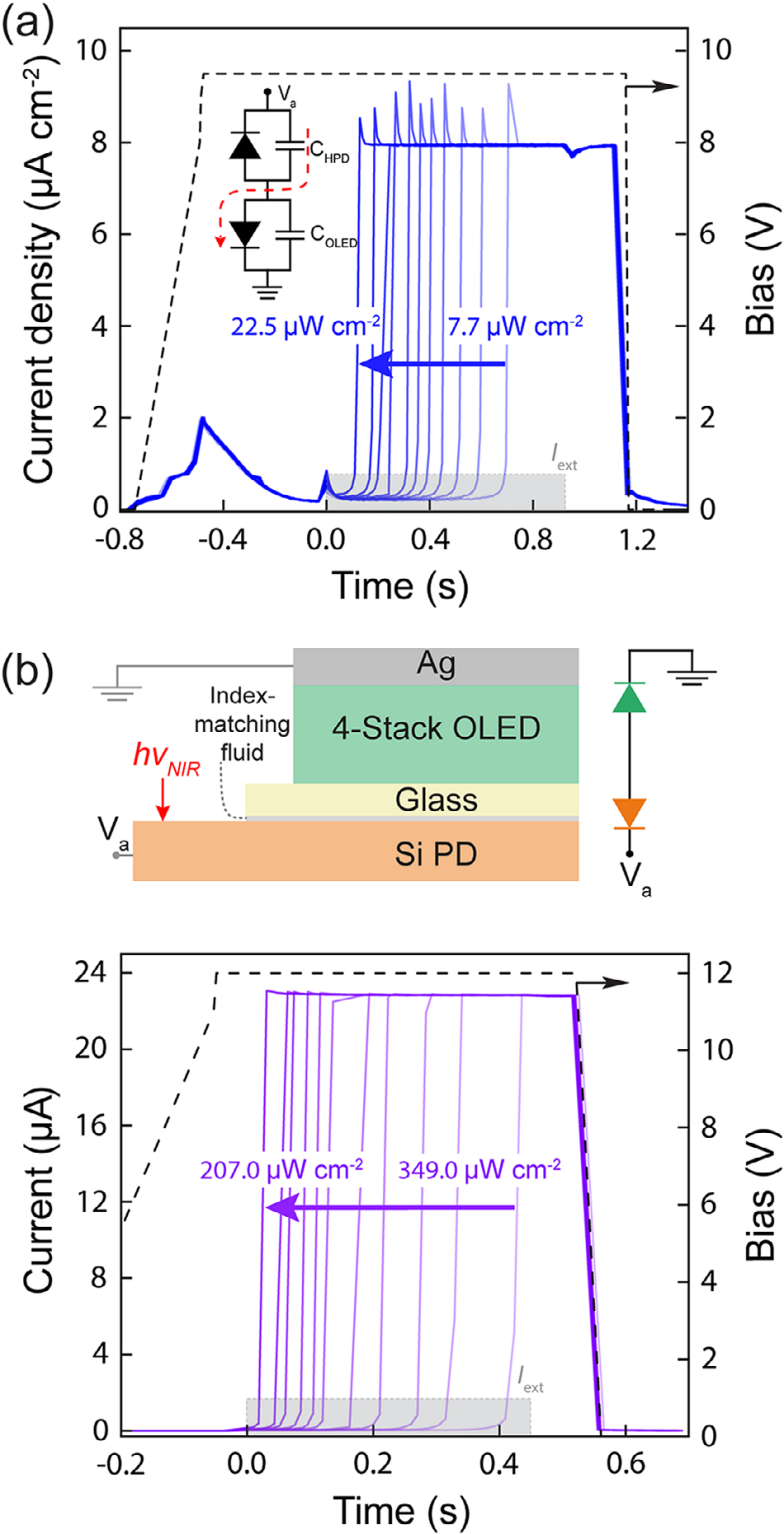
Upconverter dynamic response a) Transient response of the hybrid upconversion device to a 0.9 s illumination pulse (*I*_ext_, with duration indicated by the gray shaded rectangle; 𝜆 = 1050 nm, incident through the Si substrate) with different intensities beginning at *t* = 0. Prior to illumination, the bias is ramped up to its steady‐state value at a rate of ≈30 V s^−1^ (black dashed line corresponding to the right‐hand axis). The equivalent circuit in the inset shows the capacitance associated with the HPD and the OLED; the dashed red arrow indicates the path of the displacement current that flows when the voltage is ramped up in the dark. b) Similar transient response data (to a 0.5 s illumination pulse) collected for a component‐wise upconverter implemented by coupling a standalone 4‐stack tandem OLED to a commercial Si photodiode (Hamamatsu, S12915) with index matching fluid (*n* = 1.5) and wiring the devices together in series as illustrated in the top diagram. The active area of the photodiode is larger than the OLED, enabling illumination to be provided in the adjacent uncovered region.

**Figure** **4**a explores the dynamic response of the device to stepped illumination at varying intensities above the threshold required to trigger the jump from the low to high state. Similar to the all‐organic upconverter from Ref. [10], the transition occurs more quickly with increasing pulse intensity since initiating the process with a higher photocurrent means that fewer feedback cycles are needed to reach the upper state. A key difference, however, is that the hybrid device exhibits a current overshoot upon reaching the high state as well as a displacement‐like current when ramping up the voltage.

The fact that these features are absent for the component‐wise version of the hybrid device assembled from a standalone tandem OLED and a commercial off‐the‐shelf Si photodiode (Figure 4b) suggests that they are related to the HPD which, notably, has a much larger capacitance (≈330 nF cm^−2^) than the commercial pin photodiode (4.4 nF cm^−2^) used in the component‐wise implementation. The capacitance‐voltage measurements are provided in Supplementary Figure S3 (Supporting Information). Referring to the equivalent circuit in the inset of Figure 4a, the large HPD capacitor thus allows for a significant displacement current (red dashed line) to flow through the forward‐biased OLED while the voltage is ramped up; a negative displacement current is not observed when the voltage is ramped down because the OLED is reverse biased (the diode is off) and its capacitance is small (≈9 nF cm^−2^). Once the HPD capacitance is charged following the voltage ramp, it begins to discharge through the photodiode when the light level in the system (and thus current through the photodiode) has increased to the high state, which explains the current overshoot in Figure 4a.

### 2.4 Pixelation

**Figure 5:**
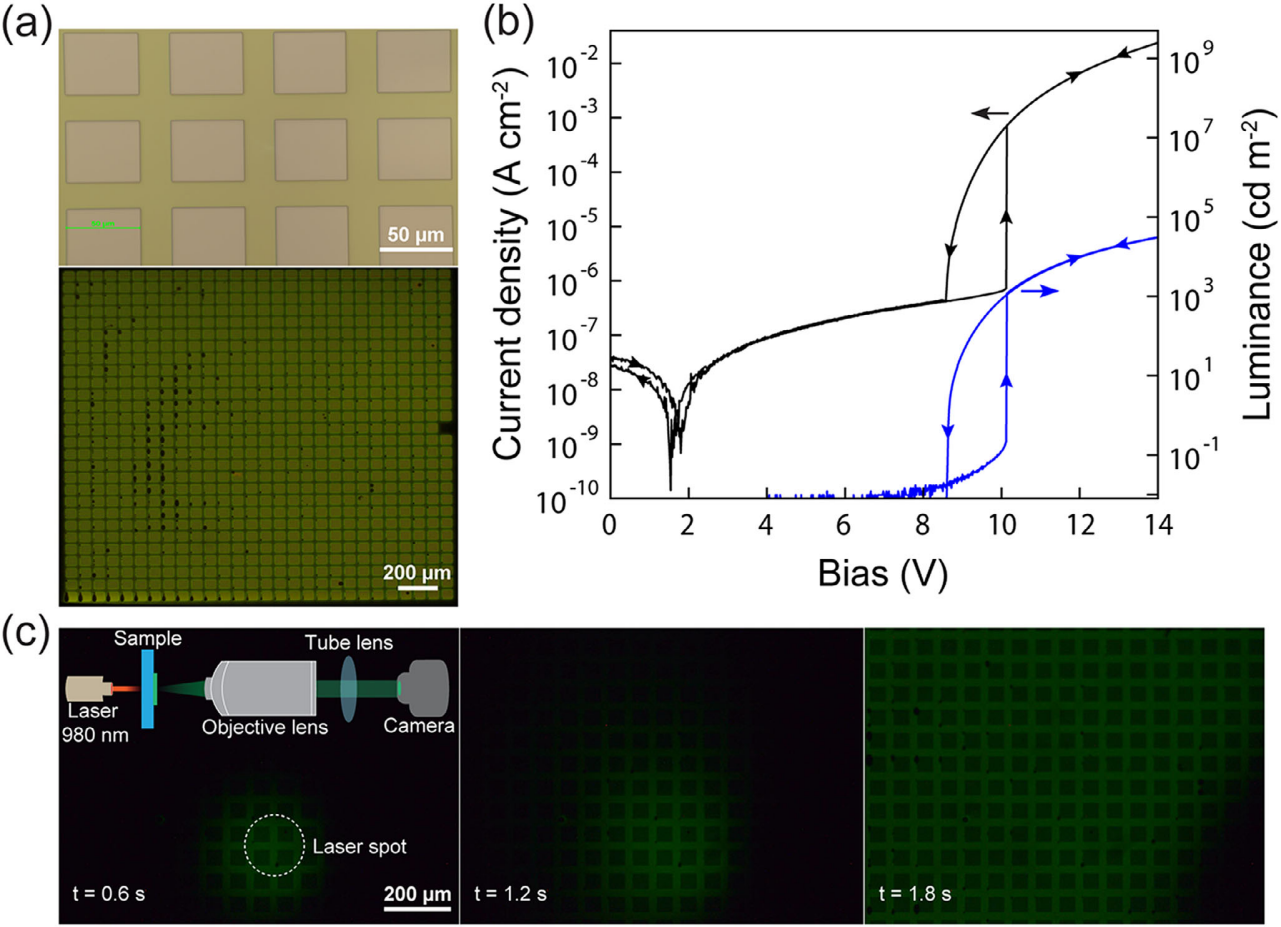
Pixelated device operation. a) Brightfield optical microscope image (top) showing the windows in the SiO_2_ layer that define each pixel when the device is lighted (bottom). All of the pixels in the device share a common cathode. b) Current‐voltage‐luminance characteristic of a pixelated device measured in the dark. c) Series of still frames extracted from Supplementary Movie S1 (Supporting Information), showing how locally triggering a group of pixels at the bottom of the array with focused illumination leads to a lateral cascade that eventually turns on the entire array. The bias is set just above the lower bound of the hysteresis window at 9.16 V in order to slow down the speed of the cascade so that it can be captured by the camera frame rate. At higher biases, the cascade occurs on a ms timescale.

Pixelation is required for imaging in bistable mode (i.e., 8 < *V*_a_ < 10 V in Figure 3a) because the positive feedback cascades laterally within a single device, causing the entire active area to light up even when only a small region is illuminated. **Figure** **5** presents an initial attempt to create a pixelated device by patterning a 28x28 array of 50x50 µm^2^ windows with a 70 µm pitch in the SiO_2_ layer (Figure 5a). The dark J‐V characteristic in Figure 5b confirms bistable operation for the array with negligible pixel‐to‐pixel variation in threshold voltage, which would otherwise manifest in multiple discrete jumps originating from different pixels transitioning at slightly different voltages^[^ ^10^ ^]^ near the nominal low‐to‐high state transition at ≈10 V. Unfortunately, when illuminated locally at the bottom of the array, the time series of images in Figure 5c (taken from Supplementary Movie S1, Supporting Information) shows that positive feedback still cascades laterally in spite of the pixelation, eventually lighting up the entire array within ≈2 s.

## 3 Discussion

The lateral cascade phenomenon can originate from optical crosstalk (i.e., waveguided electroluminescence from a bright pixel triggers its neighbor), electrical crosstalk (i.e., lateral flow of current from a bright pixel to a neighboring dark one), or a combination of both mechanisms. While optical crosstalk likely plays a role in upconverter devices fabricated on a transparent substrate such glass,^[^ ^10^ ^]^ it should be negligible for the present hybrid devices owing to strong absorption of green light by the Si. To this point, no guided modes exist in the Ag/organic/SiO_2_/Si interstitial regions between pixels owing to the high refractive index of Si, and the attenuation length for all of the leaky modes is <3 µm, which means that <0.01% of the light from an active pixel should leak laterally into its neighbor.

We therefore ascribe the cascade in Figure 5c to electrical crosstalk. Finite element simulations (Supplementary Figure S4, Supporting Information) support this conclusion, demonstrating that some of the current in an active pixel flows laterally in the Si beneath the SiO_2_ interstitial regions. Some current may also flow laterally via the conductive charge generation layers that link each OLED sub‐unit in the tandem stack. Standard pixel isolation approaches (e.g., isolation trenches) that are already used for CMOS imagers^[^ ^14^, ^15^, ^16^ ^]^ and OLED microdisplay technology^[^ ^17^, ^18^ ^]^ should be able to reduce both leakage pathways as detailed in Supplementary Figure S4 (Supporting Information).

In addition to overcoming the pixel crosstalk, it will be important to improve the sensitivity (i.e., the minimum light intensity needed to trigger the jump from the low to high state) and response speed of these devices. Shifting from the rudimentary hybrid p‐n junction used here to an optimized Si p‐i‐n junction beneath the OLED stack would increase the responsivity of the photodiode while significantly decreasing its dark current and capacitance. In this context, much of the underlying photodiode design, isolation, and control could likely be borrowed from existing CMOS image sensors,^[^ ^19^ ^]^ though in this case the photodiodes would need to be optimized for bidirectional conversion (i.e., NIR light incident from one side and visible OLED light incident from the other).

## 4 Conclusion

In summary, we have demonstrated positive feedback upconversion using a tandem OLED‐on‐Si device architecture. This device demonstrates clear bistability and a Geiger mode upconversion response to NIR illumination intensity below 1 µW cm^−2^. Compared to organic photodiode‐based implementations, this hybrid approach establishes a path to extend the upconversion spectral response further into the NIR (𝜆 = 1.1 µm demonstrated here, and potentially to 𝜆 ≈1.7 µm using InGaAs),^[^ ^6^, ^7^, ^8^ ^]^ improve sensitivity, and overcome pixel crosstalk for high resolution upconversion imaging applications. The threshold‐like response, compactness, and low power consumption of such pixelated upconverters may also prove useful for implementing the nonlinear activation function in optoelectronic multilayer neural networks that are currently being explored as accelerators for image classification and other inference tasks.^[^ ^11^ ^]^

## 5 Experimental Section

### Sample Preparation

All of the upconverter devices are fabricated on 380 µm‐thick, double side polished, 50 mm n‐doped Si wafers with 1‐10 Ω‐cm resistivity. The wafers are thermally oxidized to grow a 150 nm‐thick layer of SiO_2_. Windows are photolithographically patterned in the SiO_2_ layer to define the 0.04 cm^2^ device active areas as well as the locations of the Ohmic Ti anode contacts, which are deposited via electron beam evaporation. The substrates are cleaned with a water rinse and dried with N_2_ prior to loading into a vacuum thermal evaporator for deposition of the organic layers. No attempt is made to remove the native oxide in the device active areas during this step, as previous work has shown that it is beneficial for reducing surface recombination in organic/Si heterojunction photovoltaics.^[^ ^13^ ^]^ The organic layer stack of the full upconversion devices is as follows: n‐Si/native oxide/p‐HTM (50 nm)/4‐stack tandem green phosphorescent OLED/Ag (15 nm). The structure of the tandem OLED stack is the same as in Ref. [10] and consists of four repeat units of HTM (7 nm)/Green phosphorescent EML (20 nm)/HBL (10 nm)/ETL (10 nm)/CGL (37 nm), where EML, HBL, ETL, and CGL stand for emissive layer, hole blocking layer, electron transport layer, and charge generation layer, respectively. The CGL in the final OLED subunit is replaced by a 10 nm‐thick electron injection layer (EIL). As in Ref. [10], the materials employed in the OLED stack are proprietary, but are similar to public domain materials that yield equivalent tandem OLED performance and could readily be substituted to achieve comparable results.^[^ ^20^ ^]^ The organic stack and the Ag cathode are deposited through separate shadow masks, after which the devices are encapsulated in a N_2_‐filled glovebox using a bead of ultraviolet‐curable epoxy, desiccant, and a glass lid. The results reported here are representative of more than ten different devices fabricated and tested over the course of one year. Individual devices were tested more than 50 times during this time period and typically exhibited less than a 15% variation in dark threshold voltage and on‐state current density.

### Characterization

All experimental measurements are performed at room temperature in ambient conditions. Current‐voltage characteristics of the devices are measured using a Keysight B2912A dual source/measure unit (SMU). The voltage is swept in 0.02 V increments up and back at a rate of ≈0.1 V s^−1^. External illumination is provided with a λ = 1050 nm LED projected through a diffuser to deliver uniform intensity across the device area. The LED current is controlled by one channel of the SMU and is calibrated to deliver a known intensity at the plane of the device. In light input‐output measurements, the sweep rate of the illumination is kept low (<0.02 µW cm^−2^ s^−1^) so that it does not influence the threshold light intensity measured at a given voltage.

The photocurrent external quantum efficiency of the hybrid photodetector is measured using a laser‐driven Xe light source filtered through a monochromator. The light is chopped at a frequency of 389 Hz, and the photocurrent is detected synchronously with a lock‐in amplifier. Capacitance‐voltage measurements are carried out using a Zurich Instruments MFIA impedance analyzer.

The transient response of the devices to sudden illumination is recorded using the SMU to measure the device current in time point mode with a temporal resolution of 8 ms. A typical measurement begins by ramping the bias voltage from zero to the target value over roughly half a second before turning on the LED. The measurement cycle is repeated at a frequency of 0.4 Hz (hybrid upconverter) and 1.4 Hz (component‐wise upconverter) with a duty cycle of 80%. The initial ramp in bias voltage is necessary because rapidly turning on the voltage leads to a displacement current from the large HPD capacitance that can trigger the positive feedback cycle without any LED input, as discussed in the main text.

## Conflict of Interest

The authors declare no conflict of interest.

## Supporting information



Supporting Information

Supplementary Movie 1

## Data Availability

The data that support the findings of this study are available from the corresponding author upon reasonable request.
