# Supplementary material for: Positive Feedback Organic‐on‐Silicon Upconversion Devices
Source: Adv Sci (Weinh). 2025 Nov 9;13(5):e11468. doi: 10.1002/advs.202511468 (PMC12850483; doi:10.1002/advs.202511468)
Supplement: Supplementary file 1 — Supporting Information [file ADVS-13-e11468-s002.pdf]

SUPPLEMENTARY MATERIAL

Positive feedback organic-on-silicon upconversion devices

Raju Lampande,[1] Jon-Paul DesOrmeaux,[2] Adrian Pizano,[1] Urcan Guler[3], John W. Hamer,[2*] and Noel C. Giebink[1†]

[1]*Department of Electrical Engineering and Computer Science, University of Michigan, Ann Arbor, Michigan 48109, USA*

[2]*OLEDWorks LLC, Rochester, New York 14606, USA*

[3]*RTX Technology Research Center, East Hartford, Connecticut 06108, USA*

[*]email: jhamer@oledworks.com
[†]email: ngiebink@umich.edu

Table of contents

Note 1: Comparison of hybrid and all-organic upconverter performance

Figure S1: Upconverter measurement setup

Figure S2: Photon-to-photon upconversion efficiency

Figure S3: Capacitance of component devices

Figure S4: Finite element simulation of electrical cross-talk in pixelated devices

Supplementary Movie 1: Lateral cascade in a pixelated device

Note 1. Comparison of hybrid and all-organic upconverter performance

Table S1 below compares the performance characteristics of the hybrid upconverter from this work with the all-organic device from Ref. 10. The primary performance improvements for the hybrid device architecture are 1) its extended spectral response and 2) its reduced operating voltage. The lower photon-to-photon upconversion efficiency of the hybrid device architecture is mainly a result of operating it at a lower high-state current density and could be improved by increasing the dark threshold voltage of the device.

Table S1.

Parameter	Hybrid upconverter (this work)	All-organic upconverter (Ref. 10)
λ_{cutoff} (nm)	1100	800
$\lambda_{operating}$ (nm)	1050	750
λ_{em} (nm)	570	570
$V_{a,th}$ (V)	10.02	22.02
$I_{ext,th}$ (μW cm^{-2})	0.78 at 9.5 V	1.4 at 21.5 V
$\eta_{p\text{-}p\ (max)}$	9 at 9.8 V	192 at 22.1 V

$\lambda_{\mathrm{cutoff}}$: long wavelength edge of the spectral response range, $\lambda_{\mathrm{operating}}$: wavelength at which upconversion efficiency is measured, λ_{em}: average upconverted emission wavelength, $V_{a,th}$: dark threshold voltage, $I_{ext,th}$: minimum external light intensity required to trigger the device at the indicated operating voltage, $\eta_{\mathrm{p-p,max}}$: maximum photon-to-photon upconversion efficiency.

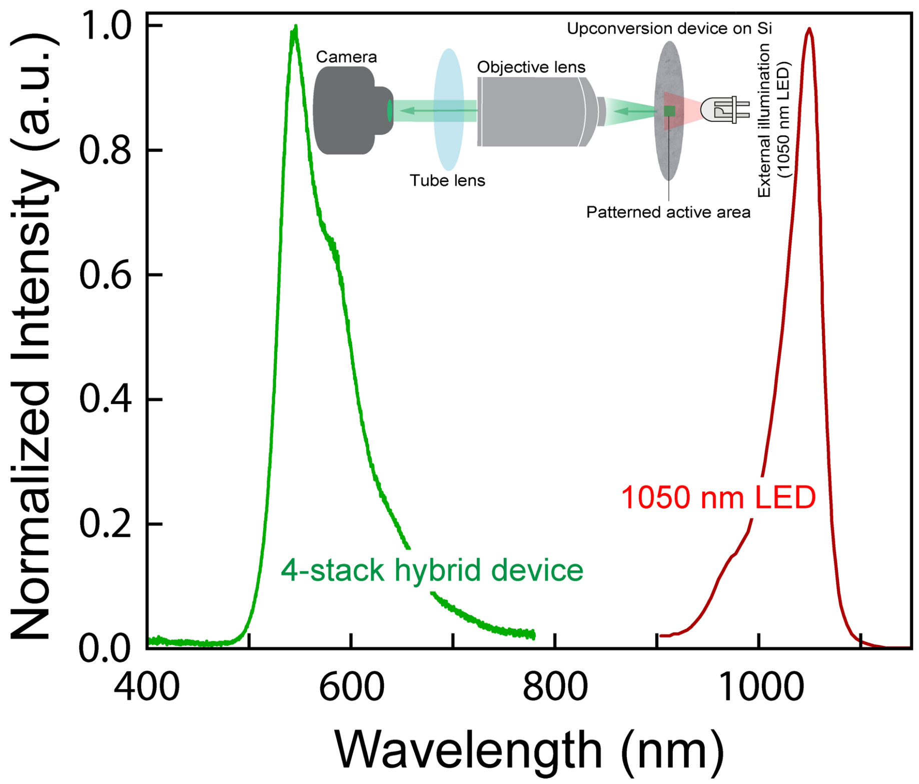

Figure S1. Electroluminescence spectrum of the fabricated four-stack hybrid upconverter device (green) and the emission spectrum of the 1050 nm LED source used for external illumination (red). The inset illustrates the measurement configuration.

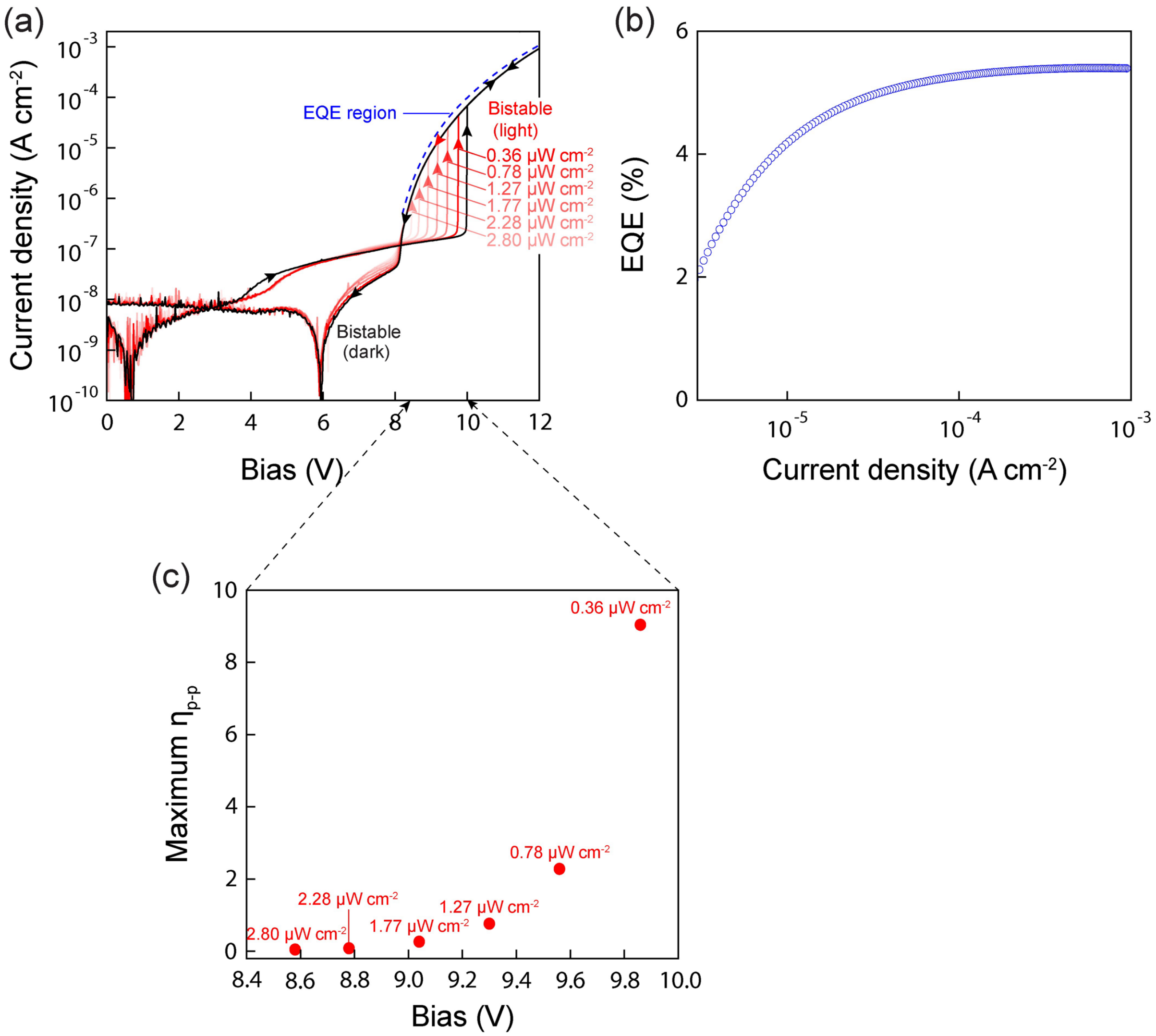

Figure S2. (a) Current density-voltage characteristic of the upconversion device reproduced from Fig. 3a for convenience. **(b)** Electroluminescence external quantum efficiency ($\eta_{\mathrm{EQE,EL}} = \eta_{\mathrm{OLED}}\eta_{\mathrm{oc}}$) measured for the high state of this device (i.e. the blue dashed region of the curves in (a)). **(c)** Maximum photon-to-photon upconversion efficiency calculated for different bias values within the bistable range of operation. The maximum upconversion efficiency is defined as the high-state output photon flux (i.e. the high state current density multiplied by $\eta_{\mathrm{EQE,EL}}$) divided by the minimum input photon flux (corresponding to the input intensities noted in red at $\lambda = 1050$ nm) required to trigger the device.

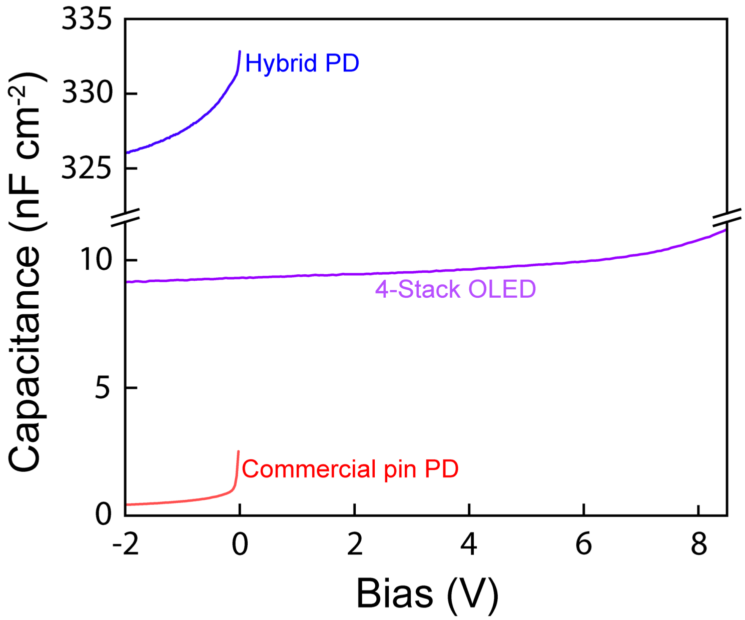

Figure S3. Capacitance-voltage characteristics measured at 1kHz for the HPD, a standalone 4-stack tandem OLED, and the commercial Si pin PD used in the component-wise upconverter implementation from Fig. 4b in the main text. The areal capacitance of the HPD is an effective value that includes both the capacitance of the actual device and the parasitic capacitance contribution from the long Ag contact trace on top of the SiO_2 layer.

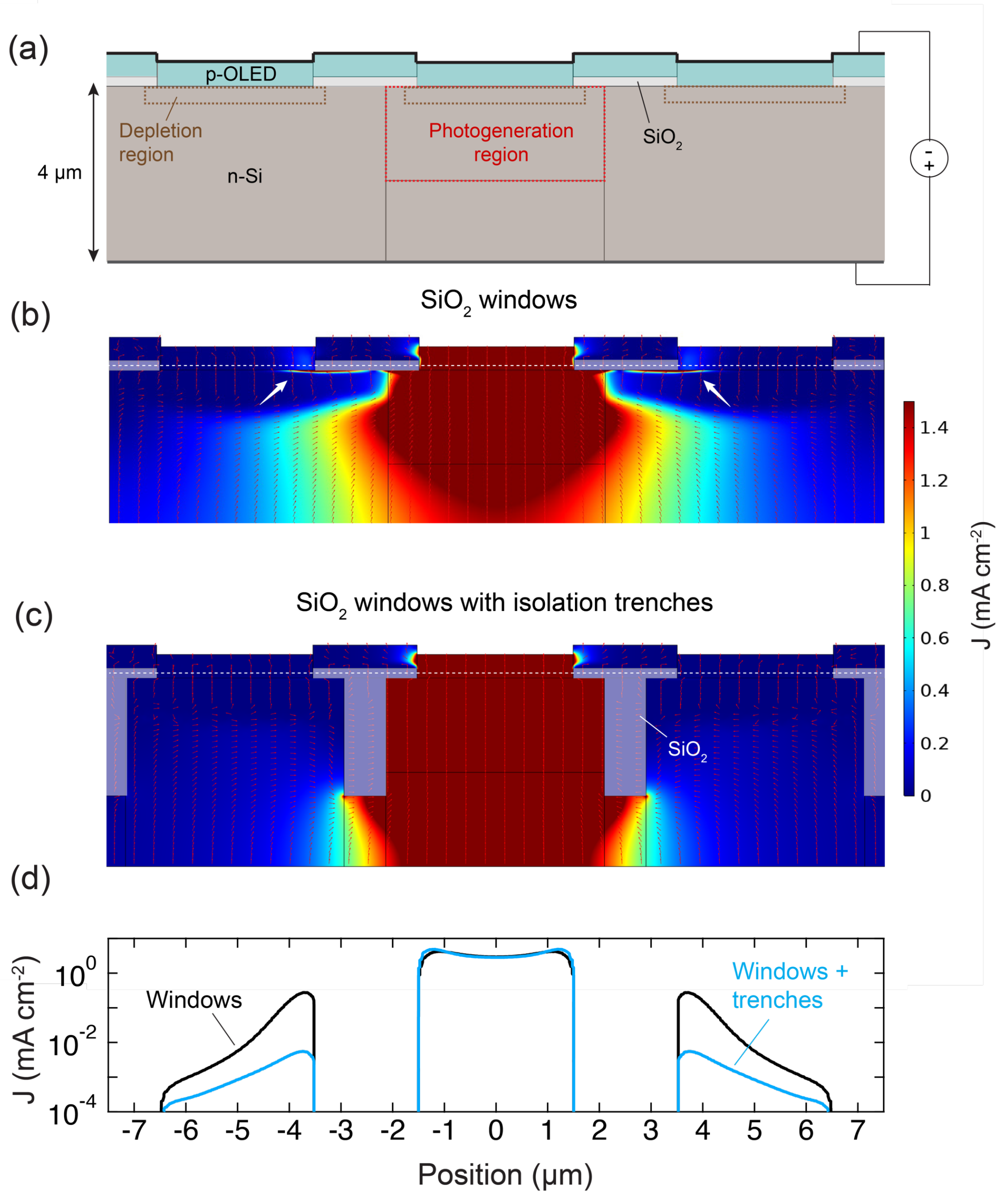

Figure S4. Finite element electrical simulations of a pixelated device. **(a)** Geometry of the simulation. The pixel dimensions are scaled down (3 μm SiO_2 windows with a 5 μm pitch) to reduce the computational load in the simulation. Accordingly, we set the minority carrier lifetime in the Si to 1 ns so that the hole diffusion length (L_p = 1 μm) decreases by roughly the same factor as the simulated device dimensions relative to experiment (i.e. both are roughly 15x smaller in the simulation than in the experiment). The band alignments and doping concentrations are maintained from Fig. 2a in the main text, and the entire OLED is treated as a 500 nm-thick layer of p-doped

HTM for simplicity. Electrons and holes are photogenerated in the dashed red box at a rate of 10^{20} $cm^{-3}s^{-1}$ to simulate absorption of green OLED light emitted by the middle pixel in its high state. The dashed brown boxes indicate the approximate extent of the depletion region in the Si underneath each pixel. **(b)** Magnitude (color map) and direction (red arrows) of the local current density simulated for the device in (a) operating at 1 V bias, which corresponds to roughly 9 V in experiment since this simulation does not include the ~8 V drop across the tandem OLED stack. Photocurrent from the active pixel flows laterally underneath the SiO_2 regions (indicated by the white arrows) and up through the near side of each adjacent low state pixel. This current originates from minority holes that are generated outside the active pixel depletion region (i.e. the region between the dashed red and brown boxes underneath the center pixel in (a)), which diffuse laterally and are collected by the neighboring pixel depletion regions. **(c)** This diffusion current can be decreased by introducing SiO_2 isolation trenches, as commonly done for CMOS imager sensors[1,2]. **(d)** Line cut of the current density flowing vertically through the plane of the OLED stack indicated by the dashed white lines in (b) and (c). Incorporating the isolation trench reduces the peak current density through the adjacent pixels by a factor of ~100.

Supplementary Movie S1.

This video shows the lateral cascade that occurs upon illuminating a group of pixels at the bottom of the frame and is the source for the still images in Fig. 5c in the main text. The experimental conditions are as detailed in the caption of Fig. 5c.

Supplementary References

1. B. J. Park *et al.*, "Deep Trench Isolation for Crosstalk Suppression in Active Pixel Sensors with 1.7 μm Pixel Pitch," *Jpn J Appl Phys*, vol. 46, no. 4S, p. 2454, Apr. 2007, doi: 10.1143/JJAP.46.2454.
2. F. Tournier *et al.*, "Pixel-to-pixel isolation by deep trench technology: Application to CMOS image sensor," in *Proc. Int. Image Sensor Workshop (IISW)*, Hokkaido, Japan , Jun. 2011, p. R5.
